# Luminescent Afterglow Behavior in the M_2_Si_5_N_8_: Eu Family (M = Ca, Sr, Ba)

**DOI:** 10.3390/ma4060980

**Published:** 2011-05-27

**Authors:** Koen Van den Eeckhout, Philippe F. Smet, Dirk Poelman

**Affiliations:** LumiLab, Department of Solid State Sciences, Ghent University, Krijgslaan 281/S1, 9000 Gent, Belgium; E-Mails: Philippe.Smet@UGent.be (P.F.S.); Dirk.Poelman@UGent.be (D.P.)

**Keywords:** persistent luminescence, afterglow, nitrido-silicates, europium, rare earths, thermoluminescence

## Abstract

Persistent luminescent materials are able to emit light for hours after being excited. The majority of persistent phosphors emit in the blue or green region of the visible spectrum. Orange- or red-emitting phosphors, strongly desired for emergency signage and medical imaging, are scarce. We prepared the nitrido-silicates Ca_2_Si_5_N_8_:Eu (orange), Sr_2_Si_5_N_8_:Eu (reddish), Ba_2_Si_5_N_8_:Eu (yellowish orange), and their rare-earth codoped variants (R = Nd, Dy, Sm, Tm) through a solid state reaction, and investigated their luminescence and afterglow properties. In this paper, we describe how the persistent luminescence is affected by the type of codopant and the choice and ratio of the starting products. All the materials exhibit some form of persistent luminescence, but for Sr_2_Si_5_N_8_:Eu,R this is very weak. In Ba_2_Si_5_N_8_:Eu the afterglow remains visible for about 400 s, and Ca_2_Si_5_N_8_:Eu,Tm shows the brightest and longest afterglow, lasting about 2,500 s. For optimal persistent luminescence, the dopant and codopant should be added in their fluoride form, in concentrations below 1 mol%. A Ca_3_N_2_ deficiency of about 5% triples the afterglow intensity. Our results show that Ba_2_Si_5_N_8_:Eu(,R) and Ca_2_Si_5_N_8_:Eu(,R) are promising persistent phosphors for applications requiring orange or red light.

## 1. Introduction

Some luminescent materials continue emitting light for minutes or hours after removal of the excitation. This phenomenon, known as **persistent luminescence**, is sometimes undesired, as it limits the use of these materials in applications that require high switching speeds, such as LEDs or information displays. On the other hand, the very long lifetime of the emission without the need of a constant energy input opens up possibilities in many other fields such as, but not limited to, emergency signage [1], traffic safety, medical imaging [2], dials and displays, and decoration.

In 1996, the discovery of bright green afterglow in SrAl_2_O_4_:Eu,Dy [3] boosted the search for new and better persistent luminescent materials. Today, several efficient persistent phosphors are known and described [4], mainly aluminates such as Sr_4_Al_14_O_25_:Eu,Dy [5] and (di)silicates like Sr_2_MgSi_2_O_7_:Eu,Dy [6]. Most of these emit light in the blue or green region of the visible spectrum. The lack of potent red-emitting afterglow phosphors, which are desired for emergency signage and medical imaging [2], is caused by two reasons:

- Firstly, the sensitivity of the human eye is low in the orange-to-red region of the visible spectrum. This effect is even more dramatic at low light conditions, which are typical for persistent luminescent applications. Below 1 cd/m², in the mesopic and scotopic regime, the eye sensitivity shifts to shorter wavelengths, a phenomenon known as the Purkinje effect. This makes it very hard to detect red light in dark environments. Because of this, a potential red-emitting persistent phosphor needs a much higher radiance in order to achieve the same apparent brightness as a green- or blue-emitting one [7].
- Secondly, the large majority of persistent luminescent materials are based on divalent europium as luminescent centre. The position of the broad emission spectrum of Eu^2+^ strongly depends on the interaction with the host compound [8]. However, in the aforementioned aluminates and silicates, and by extension in almost all oxides, it is difficult to achieve a red-shift that is large enough to obtain red emission from divalent europium.

The first problem is inherent to human vision and cannot be solved, but the second one can be addressed by turning to other host materials such as sulfides [9] or, in the present case, nitrides.

During the past few years, the europium-doped alkaline earth nitrido-silicates M_2_Si_5_N_8_:Eu (M = Ca, Sr, Ba) have gained popularity as wavelength conversion phosphors in white LEDs, because of their large chemical and thermal stability and high quantum efficiency [10]. Additionally, promising afterglow properties have been reported in some of these materials [11,12,13,14]. Previously, we have shown that Ca_2_Si_5_N_8_:Eu has an orange afterglow, which can be greatly enhanced by Tm codoping [15]. To study the influence of the lattice cations on the persistent luminescence we have extended this investigation to include Sr_2_Si_5_N_8_:Eu and Ba_2_Si_5_N_8_:Eu, which have emission spectra very similar to Ca_2_Si_5_N_8_:Eu. As we describe in this paper, the afterglow is not only influenced by codoping, but also depends on the starting materials and the dopant and codopant concentrations.

## 2. Experimental

All powders were prepared using a solid state reaction at 1,400 °C for 3 hours, under reducing atmosphere of forming gas (90% N_2_, 10% H_2_). The host material was made by mixing appropriate amounts of M_3_N_2_ (99%, Alfa Aesar for Ca_3_N_2_ and Cerac for Ba_3_N_2_ and Sr_3_N_2_) and α-Si_3_N_4_ (99.85%, Alfa Aesar). Dopants (europium) and codopants (rare earths, R) were added to the starting mixture in oxide (R_2_O_3_) or fluoride (RF_3_) form (typically 99.9%, Alfa Aesar). Unless mentioned otherwise, the powders were prepared with 1 mol% of Eu and R (*i.e*., 1% substitution of the alkaline earth ions). All materials were weighed, ground and mixed under a protective N_2_ atmosphere in a glove box prior to the solid state reaction.

Crystallographic phases of the obtained powders were checked using X-ray diffraction (XRD) with a Bruker D5000 θ–2θ diffractometer using Cu-Kα radiation and compared with literature data [16,17]. The band gap was estimated through diffuse reflection measurements using a Varian Cary 500 spectrophotometer equipped with an integrating sphere.

The photoluminescent properties (emission, excitation) of the samples were measured with a fluorescence spectrometer (FS920, Edinburgh Instruments). Afterglow decays were measured with both the aforementioned spectrometer and a calibrated photometer (ILT 1700, International Light Technologies). Microsecond decay measurements were performed using a pulsed nitrogen laser setup (λ_exc_ = 337 nm, pulse length 800 ps, repetition rate 1 Hz) and a 1024-channel intensified CCD (Andor Technology) attached to a 0.5 m Ebert monochromator.

Thermoluminescent curves were obtained at Delft University of Technology, The Netherlands. The samples were exposed to two minutes of 300 nm irradiation at room temperature and the emission during heating was monitored using an OceanOptics QE65000 CCD-based spectrometer.

## 3. Results and Discussion

### 3.1. Structural Properties

The M_2_Si_5_N_8_ compounds crystallize in two different structure types. The orthorhombic Sr_2_Si_5_N_8_ and Ba_2_Si_5_N_8_ are isostructural, both having space group Pmn2_1_ [17]. In contrast, Ca_2_Si_5_N_8_ has a monoclinic structure of space group Cc [16]. Because of this, the properties of the different compounds cannot be readily compared. For example, the position of the emission spectrum of M_2_Si_5_N_8_:Eu^2+^ does not shift continuously by changing the alkaline earth ion. Also the excitation spectrum of Ca_2_Si_5_N_8_:Eu differs significantly from those of Sr_2_Si_5_N_8_:Eu and Ba_2_Si_5_N_8_:Eu [10].

Upon (co)doping, the rare earth ions occupy the lattice sites of the alkaline earth cations in the host. Due to the different size of the rare earth ions, the lattice parameters slightly change upon (co)doping. For example, in Ca_1.94_Eu_0.02_Tm_0.04_Si_5_N_8_ (where Eu^2+^ is somewhat larger than Ca^2+^, and Tm^3+^ is notably smaller [18]) the volume of the unit cell is reduced by around 1.7% compared to the undoped compound. The type of dopant or codopant has no significant influence on the crystal structure.

Diffuse reflection spectra on undoped samples (not shown) were performed to verify the band gap of the materials. Ca_2_Si_5_N_8_ has the largest band gap (around 250 nm), followed by Sr_2_Si_5_N_8_ (265 nm) and Ba_2_Si_5_N_8_ (280 nm). These values are the same as found by Li *et al.* [10], except for Ba_2_Si_5_N_8_ where they found a band gap of 270 nm.

### 3.2. Photoluminescent Properties

**Figure 1:**
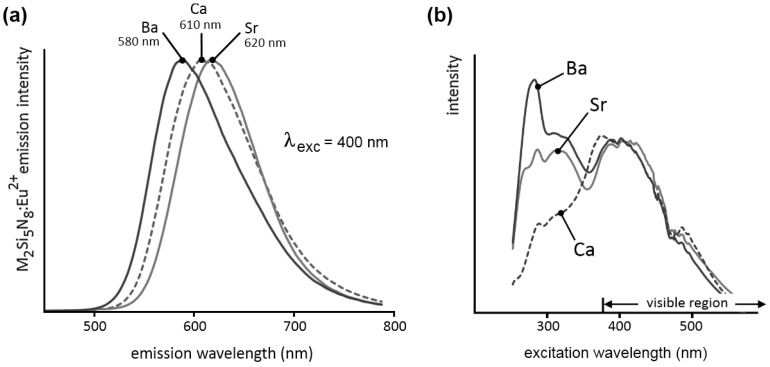
**(a)** Emission and **(b)** excitation spectra of M_2_Si_5_N_8_:Eu (M = Ca,Sr,Ba). The emission spectra were recorded for λ_exc_ = 400 nm. The excitation spectra were recorded for the emission at 610 nm for M = Ca, 620 nm for M = Sr and 580 nm for M = Ba.

Figure 1a shows the steady state emission spectra (emission spectra recorded during excitation) of Ca_2_Si_5_N_8_:Eu, Sr_2_Si_5_N_8_:Eu and Ba_2_Si_5_N_8_:Eu. In all three cases, the spectrum consists of one broad Eu^2+^-based band (FWHM of about 100 nm). The broadness of the emission can be explained by the existence of two similar cation sites in the host lattice [16,17]. Since the europium ions occupy these cation sites, and the position of the emission band is closely related to the europium surroundings, the spectrum will consist of two bands, in this case mostly overlapping [10,11]. The emission maximum shifts from 580 nm (yellowish orange) for M = Ba, over 610 nm (orange) for M = Ca, to 620 nm (reddish orange) for M = Sr. It could be expected that the position of the emission maximum shifts continuously when changing the cation from Ca over Sr to Ba. However, Ca_2_Si_5_N_8_:Eu does not follow this trend because of its different crystal structure compared to Sr_2_Si_5_N_8_:Eu and Ba_2_Si_5_N_8_:Eu. For concentrations below 5%, the wavelength position of the maxima is in all powders only slightly dependent on the europium concentration [10].

The steady state excitation spectra are given in Figure 1b. These are very broad, and extend well into the visible region, up to 550 nm. On the short wavelength side, the samples show exciton absorption in the region just below the band gap. The remaining bands can be attributed to the 4f^7^ → 4f^6^5d^1^ transition in the Eu^2+^ ions, with a maximum in a broad region around 400 nm.

### 3.3. Afterglow

**Figure 2:**
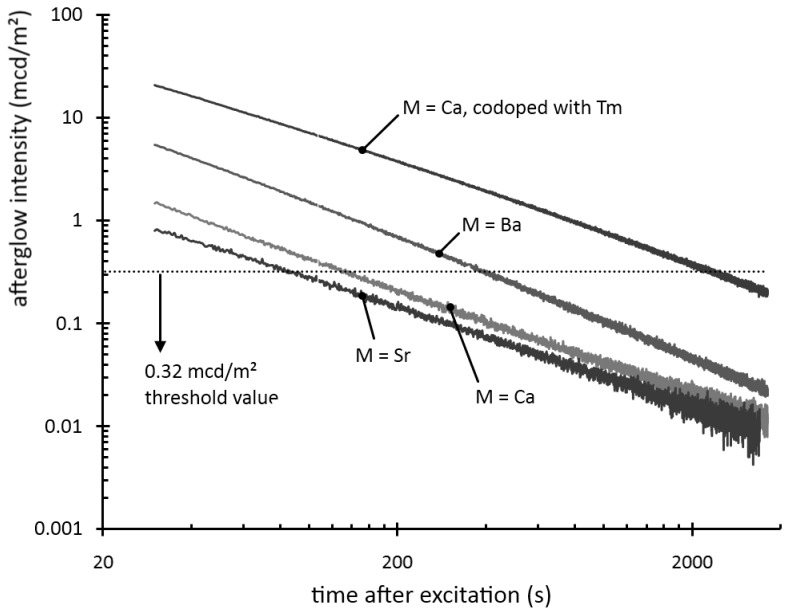
Decay of the afterglow intensity after 1 min excitation with a Xe arc lamp at 1000 lux.

After excitation, all materials show some form of persistent luminescence. Figure 2 shows the afterglow intensity as a function of time after 1 minute excitation with an unfiltered xenon arc lamp at 1000 lux. The decay curves follow straight lines in a log-log plot, implying that they can be modeled by a power law with negative scaling exponent. In other words, the decay is very fast initially, but slows down over time. This type of behavior is typical for most persistent luminescent materials.

The afterglow duration can be defined as the time between the end of the excitation and the moment when the afterglow intensity drops below 0.32 mcd/m², about 100 times the sensitivity of the human eye (a threshold value often used in industrial standards [7,19]). The afterglow duration is longest for Ba_2_Si_5_N_8_:Eu, around 400 seconds, followed by Ca_2_Si_5_N_8_:Eu, around 150 seconds. Sr_2_Si_5_N_8_:Eu has a very short afterglow duration of 80 seconds. This rapid afterglow decay is an advantage, since Sr_2_Si_5_N_8_:Eu is a popular conversion phosphor in GaN or InGaN-based white LEDs, where a long afterglow is undesired [20].

**Figure 3:**
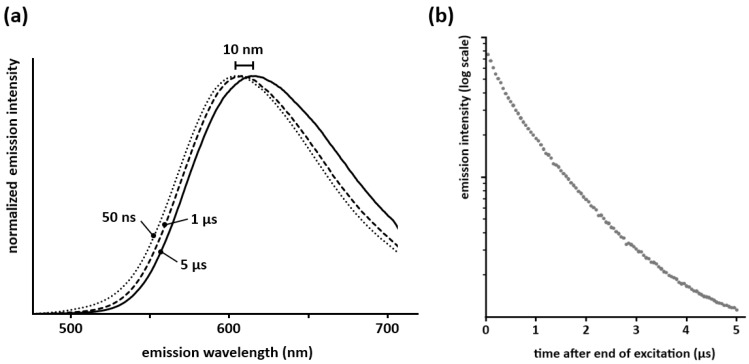
**(a)** Spectra of the persistent emission 50 ns, 1 µs and 5 µs after a pulsed 337 nm excitation (integration time of 50 ns for each spectrum, normalized for an easier comparison of the shape); **(b)** Integrated emission intensity of Ca_2_Si_5_N_8_:Eu during the first microseconds after the end of the excitation (λ_exc_ = 337 nm).

The afterglow spectra of Ca_2_Si_5_N_8_:Eu and Ba_2_Si_5_N_8_:Eu are red-shifted about 5–10 nm compared to the steady state spectra (Figure 3a; for Sr_2_Si_5_N_8_:Eu the afterglow was too weak to measure this with sufficient accuracy. A similar red-shift in the persistent spectrum is also seen in Ca_2_SiS_4_:Eu,Nd [21] and CaAl_2_Si_2_O_8_:Eu [22].

Measurements of the decay behavior in the first moments after the end of the excitation show that this red-shift occurs in the first 5–10 µs after the end of the excitation (Figure 3a), after which the spectrum remains unchanged during the remainder of the afterglow. This means that both crystallographic sites available for europium take part in the persistent luminescence. If only one of the sites showed persistent luminescence, the decay time of the other would be determined solely by the lifetime of Eu^2+^ (around 1 µs [23]), and the persistent emission spectrum would reach its final position on a much faster timescale than the observed 5–10 µs. Secondly, the integrated emission intensity in the first microseconds after the excitation does not show a clear exponential component with a lifetime of 1 µs (Figure 3b), which would be expected if one of the sites did not take part in the persistent luminescence. Translating these observations to a defect model is not straightforward and remains somewhat speculative. A first possibility is formed by defects (which serve as trap state) being proximate to the europium ions, thus altering the local environment. This could then lead to the observed red-shift in the afterglow spectrum. Another possibility could be the affinity for traps to be dominantly coupled to only one of the europium sites. Differences in energy transfer behavior between both sites for the steady state emission and the afterglow might occur also, although this is less likely given that the steady state emission spectrum of Ca_2_Si_5_N_8_:Eu is invariable for changes in excitation wavelength or intensity. Clearly more in-depth research is required to explain this observation.

### 3.4. Rare Earth Codoping

In many persistent luminescent materials the afterglow can be greatly influenced by adding traces of various rare earths. The most notable example is SrAl_2_O_4_:Eu,Dy, where the presence of Dy codoping increases the afterglow brightness by almost two orders of magnitude [3]. The influence of each rare earth differs for every host material. To optimize the afterglow, Dy is the most common codopant, closely followed by Nd. However, other choices are possible for specific hosts, such as Tm, Ho, Y or Ce [4].

The reason behind this positive (or negative) influence on the afterglow is the subject of an ongoing debate. Most authors agree that the codopant ions influence the charge carrier traps in the material, which are responsible for the persistent luminescence. However, some researchers believe the rare earth ions merely influence the depth of pre-existing traps in the host crystal, such as oxygen vacancies (for example, Clabau *et al.* [19]), while others assume that they act as electron traps themselves (such as Dorenbos [24] and Aitasalo *et al.* [25]). In the former case, the trap depth is related to the ionization potential of the different codopant ions [19]. In the latter case, the 4f^7^ ground state levels of the doubly ionized rare-earths (trivalent codopant with a trapped electron) determine the depth of the traps [24].

**Figure 4:**
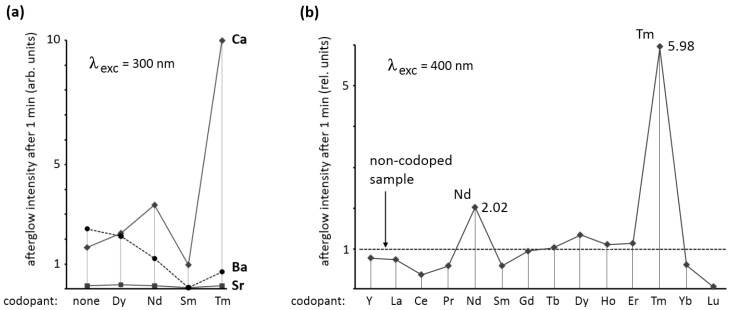
**(a)** Afterglow intensity of M_2_Si_5_N_8_:Eu,R (M = Ca,Sr,Ba) 1 min after excitation with 300 nm light, as a function of the codopant; **(b)** Afterglow intensity of Ca_2_Si_5_N_8_:Eu,R 1 min after excitation with 400 nm light, as a function of the codopant.

To study the influence of rare earth ions on M_2_Si_5_N_8_:Eu, we codoped samples with 1% of dysprosium, neodymium, samarium or thulium, and compared the persistent luminescence with the material without codoping. This codoping has no effect on the emission spectrum. Figure 4a shows the afterglow intensity 1 minute after excitation with 300 nm (violet) light. As can be seen, the codopant strongly influences the afterglow in Ca_2_Si_5_N_8_:Eu,R and Ba_2_Si_5_N_8_:Eu,R. For Sr_2_Si_5_N_8_:Eu,R no afterglow of importance could be achieved with any of the codopants. For Ba_2_Si_5_N_8_:Eu,R codoping with dysprosium does not considerably influence the afterglow. The other codopants have a negative effect on the afterglow in Ba_2_Si_5_N_8_:Eu,R.

In Ca_2_Si_5_N_8_:Eu,R a strong influence of the codopant is seen. Dysprosium and neodymium enhance the afterglow intensity considerably, while samarium reduces it by more than 40%. The most spectacular effect is obtained with thulium codoping. In this case, the afterglow intensity after one minute is nearly six times as high as in the non-codoped sample. Figure 4b shows the effect of all rare earth codopants (including yttrium, but excluding promethium) on the afterglow in Ca_2_Si_5_N_8_:Eu,R (λ_exc_ = 400 nm) proving again that thulium codoping is the best choice for persistent luminescence in this host. The afterglow duration after 1 min excitation with a Xe arc lamp at 1000 lux is around 2,500 s (Figure 2). The other rare earths, except for neodymium and dysprosium, have a negative or no effect on the afterglow intensity.

### 3.5. Thermoluminescence

The charge carrier traps, which are presumably created or affected by the codopant ions, can be investigated by thermoluminescence (TL) experiments. To collect a TL spectrum, the sample is excited at low temperature, after which it is heated (with a fixed heating rate β). During heating, the light emission is monitored, resulting in one or more broad peaks (‘glow peaks’) in an intensity-*vs*-temperature diagram. In this way an estimate of the trap depths can be made, since deeper traps require more thermal energy to be emptied, and hence will cause peaks at higher temperatures. It is usually assumed that the ideal trap depth for persistent luminescence at room temperature is about 0.65 eV [4], corresponding to glow peaks around 70–100 °C, depending on the heating rate.

**Figure 5:**
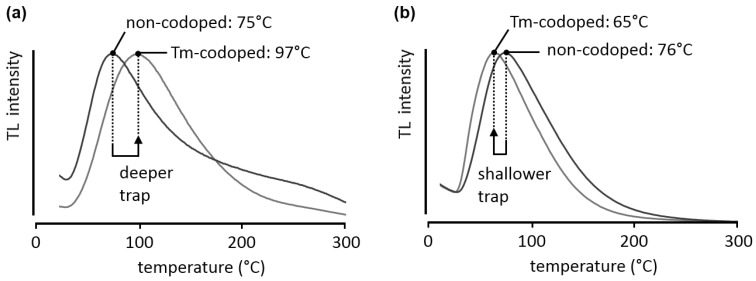
Normalized TL glow curves for **(a)** Ca_2_Si_5_N_8_:Eu and Ca_2_Si_5_N_8_:Eu,Tm; **(b)** Ba_2_Si_5_N_8_:Eu and Ba_2_Si_5_N_8_:Eu,Tm. The samples were excited at room temperature for 2 minutes, at an excitation wavelength of 300 nm. The heating rate β was 2.5 °C/min.

Figure 5a compares the glow curves obtained during a TL measurement for Ca_2_Si_5_N_8_:Eu and the thulium-codoped variant, after 300 nm excitation at room temperature for 2 minutes, with a heating rate β of 2.5 °C/min. The curves were analyzed with the *TL Glow Curve Analyzer* software [26]. Nevertheless, care should be taken when analyzing these glow curves. The broadness of the peaks, especially on the high-temperature side, indicates that a single first- or second-order trap description is insufficient and multiple traps or trap distributions might be present. Therefore, the trap depths presented here are only indicative. In both the non-codoped and the thulium-codoped case the shape of the peak is similar, suggesting that the same trap is responsible for the afterglow. However, upon codoping with thulium the peak location shifts to higher temperatures, around 100 °C, indicating that the trap depth has increased to a more suitable depth for persistent luminescence. Assuming general order kinetics, the trap depth is estimated to shift from 0.87 eV to 0.91 eV upon codoping.

To illustrate that the same codopant can have different effects in different host crystals, the glow curves for Ba_2_Si_5_N_8_:Eu and Ba_2_Si_5_N_8_:Eu,Tm are shown in Figure 5b. In this case, the glow peak shifts to lower temperatures, in other words, the charge carrier trap becomes shallower (0.71 eV in the non-codoped case, 0.68 eV in the thulium-codoped sample, again assuming general order kinetics).

### 3.6. Influence of the Starting Materials

The afterglow intensity of the samples is not only influenced by the codopants, but also by the starting materials chosen during the preparation. For example, when the dopant and codopant ions are added to the starting mixture in their fluoride form (RF_3_), the afterglow 10 minutes after excitation is over 12 times brighter than if oxides (R_2_O_3_) are used. This is most probably due to the more favorable chemical properties of fluorides compared to oxides, for example their lower melting point. This makes it easier for the materials to diffuse into the host crystal during the preparation. XRD measurements show an increased crystallinity when using fluorides, indicating that the fluorine also acts as a flux material during the reaction. EDX investigations of Ca_2_Si_5_N_8_:Eu,Tm have revealed the formation of undesired micron-sized Tm- and O-rich aggregates upon preparation with oxides (not shown). Analysis of these aggregates indicates that they contain comparable concentrations of Tm, Si and N, and almost no Ca. This suggests the formation of the non-luminescent TmSiO_2_N phase. When using fluorides some Tm-rich aggregates were still formed, but in a lower concentration. Here, the equal concentrations of Ca and Tm and the hexagonal structure of the aggregates suggest the formation of CaTmSi_4_N_7_. No fluorine was encountered in any of the samples.

A second effect of the starting materials is of a stoichiometric nature. A 5% deficiency of Ca_3_N_2_ in the starting mixture for Ca_2_Si_5_N_8_:Eu,Tm increases the afterglow intensity nearly threefold. This might be due to the fact that the dopant and codopant ions are located at Ca-sites. Hence an increase of Ca-vacancies, caused by the Ca_3_N_2_ deficiency, might facilitate the incorporation of rare earth ions in the host, because of charge compensation effects. However, as noted above, the nature and location of the traps that lead to persistent luminescence is still debated, and further research is necessary to study the incorporation of the rare earths and their influence on the afterglow. At even higher deficiencies of Ca_3_N_2_, the brightness of the persistent luminescence decreases again. XRD measurements show that a (non-luminescent) α-Si_3_N_4_ phase remains in these samples.

### 3.7. Dopant and Codopant Concentration

**Figure 6:**
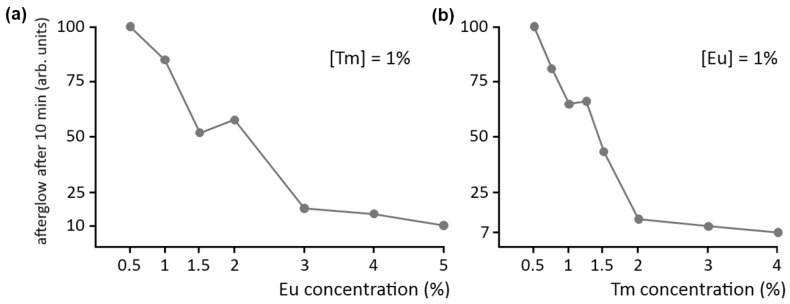
Influence of **(a)** dopant and **(b)** codopant concentration on the afterglow intensity of Ca_2_Si_5_N_8_:Eu,Tm.

A final point of interest concerns the concentration of the dopant and codopant ions in the host crystal. As with normal fluorescence, a high concentration of luminescent centers is usually undesired in persistent luminescence, due to concentration quenching. This is confirmed by Figure 6a, showing the afterglow intensity in Ca_2_Si_5_N_8_:Eu,Tm after 10 minutes as a function of Eu concentration, where the Tm codopant concentration is kept at 1%. Clearly, it is best to keep the dopant concentration below 1%.

The afterglow brightness as a function of codopant concentration is shown in a similar fashion in Figure 6b, where the Eu concentration is kept constant at 1%. The effect is even more dramatic here: a Tm concentration of 2% reduces the afterglow intensity to 10% of the value at a concentration of 0.5%. Previous SEM and EDX investigations have revealed the formation of (non-luminescent) Tm-rich aggregates in the material when the concentration becomes too high [15]. This undesired phenomenon can be partially countered by increasing the preparation duration and temperature. Still, a Tm concentration above 1% is undesired for persistent luminescence in Ca_2_Si_5_N_8_:Eu,Tm.

## 4. Conclusions

The M_2_Si_5_N_8_ (M = Ca,Sr,Ba) family of materials was synthesized using a solid state reaction, doped with Eu as luminescent centers, and codoped with Nd, Dy, Sm and Tm. All samples show persistent luminescence, but for all M = Sr samples this is very weak. The afterglow spectra are red-shifted by about 5–10 nm compared to the steady state spectrum. In Ba_2_Si_5_N_8_:Eu the afterglow lasts about 400 s, which can be slightly increased by Dy codoping. Ca_2_Si_5_N_8_:Eu,Tm shows by far the brightest and longest afterglow, lasting over 2,500 s. Thermoluminescence measurements indicate that the addition of Tm in Ca_2_Si_5_N_8_:Eu deepens the relevant charge trap to a more suitable depth.

For optimal persistent luminescence, the dopant and codopant should be added to the starting mixture in their fluoride form, in concentrations not higher than 1 mol%. During the preparation, a deficiency of Ca_3_N_2_ of about 5% compared to the stoichiometric amount triples the intensity of the afterglow.
